# Comparative Molecular Dynamics Study of 19 Bovine Antibodies with Ultralong CDR H3

**DOI:** 10.3390/antib14030070

**Published:** 2025-08-13

**Authors:** Olena Denysenko, Anselm H. C. Horn, Heinrich Sticht

**Affiliations:** 1 Bioinformatics, Institute of Biochemistry, Friedrich-Alexander-Universität Erlangen-Nürnberg (FAU), 91054 Erlangen, Germany; olena.denysenko@fau.de (O.D.); anselm.horn@fau.de (A.H.C.H.); 2 Erlangen National High Performance Computing Center (NHR@FAU), Friedrich-Alexander-Universität Erlangen-Nürnberg (FAU), 91058 Erlangen, Germany

**Keywords:** bovine antibody, ultralong CDRH3, knob domain, protein structure, molecular dynamics

## Abstract

**Background/Objectives**: Cows produce antibodies with ultralong CDRH3 segments (ulCABs) that contain a disulfide-stabilized knob domain. This domain is connected to the globular core of the antibody by a β-strand stalk. In the crystal structures, the stalk protrudes from the core in an extended conformation and presents the knob at its distal end. However, the rigidity of this topology has been questioned due to the extensive crystal packing present in most ulCAB crystal structures. To gain more insight into the dynamics of ultralong CDRH3s, we performed a comparative molecular dynamics (MD) study of 19 unique ulCABs. **Methods**: For all 19 systems, one-microsecond MD simulations were performed in explicit solvent. The analyses included an investigation of the systems’ conformational stability and the dynamics of the knob domain as well as an energetic analysis of the intramolecular knob interactions. **Results**: The simulations show that the extended stalk–knob conformation observed in the crystal structures is not preserved in solution. There are significant differences in the degree of knob dynamics, the orientations of the knobs, the number of flexible stalk residues, and the frequency of the motions. Furthermore, interactions between the knob and the light chain (LC) of the ulCABs were observed in about half of the systems. **Conclusions**: The study reveals that pronounced knob dynamics is a general feature of ulCABs rather than an exception. The magnitude of knob motions depends on the system, thus reflecting the high sequence diversity of the CDRH3s in ulCABs. The observed knob–LC interactions might play a role in stabilizing distinct knob orientations. The MD simulations of ulCABs could also help to identify suitable knob fragments as mini-antibodies by suggesting appropriate truncation points based on flexible sites in the stalks.

## 1. Introduction

Antibodies are a crucial component of the adaptive immune system in vertebrates, providing the diversity needed to recognize a wide range of antigens. Conventional antibodies consist of heavy chains (HCs) and light chains (LCs), with each chain containing constant and variable domains. The variable domains form the antigen-binding site, which contains short, hypervariable loops termed complementarity-determining regions (CDRs). Each heavy chain contains three CDRs (H1, H2, H3), and each light chain has three corresponding regions (L1, L2, L3). The H3 region of the heavy chain is the most variable CDR and plays a significant role in determining the affinity and specificity of antigen recognition [1]. The diversity of the CDRs is achieved through the genetic mechanisms of the adaptive immune system. Vertebrates utilize a combination of variable (V), diversity (D), and joining (J) gene segments that undergo recombination. This V(D)J recombination, coupled with somatic hypermutation, leads to considerable variation [2].

CDRs are of limited length in most species. For example, the most variable CDRH3 in humans typically has a length of 4–20 amino acids and rarely exceeds 25 residues [3]. By contrast, cows have a subgroup of antibodies with an ultralong CDR-H3 (ul-CDRH3) consisting of ~40–70 amino acids [4,5]. Antibodies with ul-CDRH3s are particularly effective for targeting cryptic viral epitopes, making them suitable for antiviral applications.

**Figure 1:**
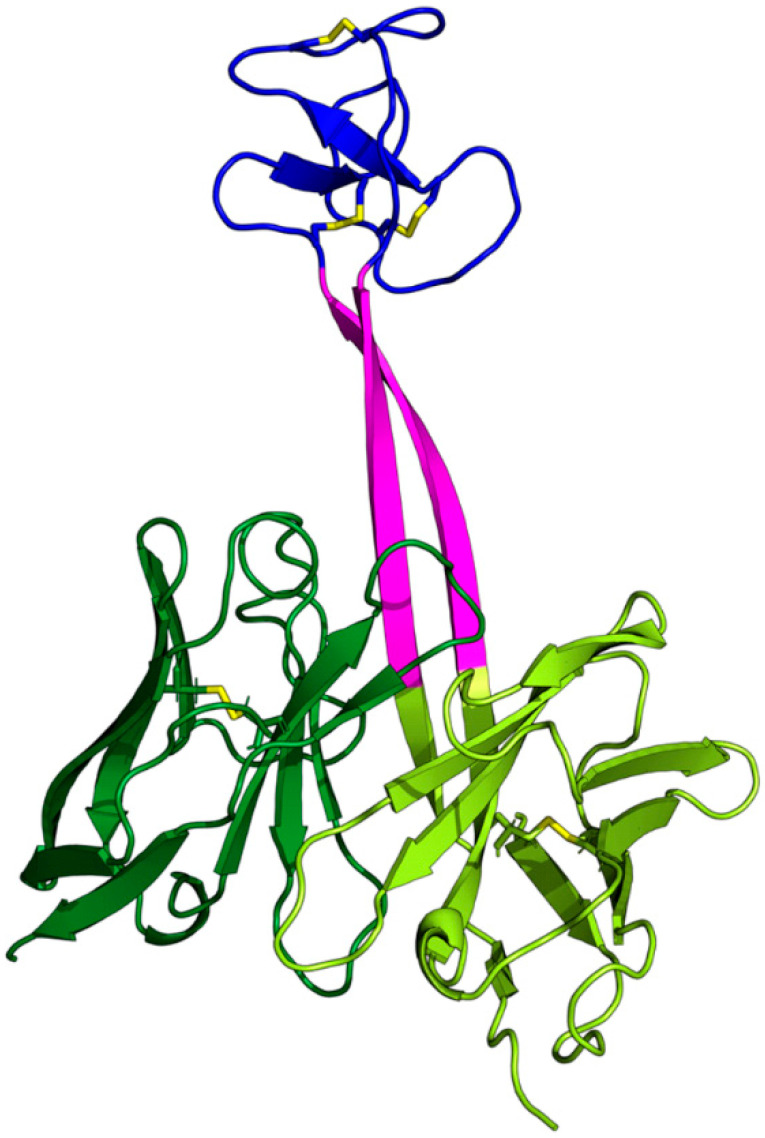
The structure of the Fv part of ulCAB BLV1H12 (PDB-ID: 4k3d [7]) illustrating the domain topology of ulCABs. The light chain (dark green) exhibits a structure similar to the LCs of other antibodies. The heavy chain consists of a globular part (light green) with an unusually long CDRH3 composed of a stalk (magenta) and a knob domain (blue). Disulfide bonds are shown as yellow sticks. The entity formed by the globular parts of the HC (light green) and LC (dark green) was termed ‘core domain’ in the present study.

Approximately 30 experimental structures of bovine antibodies with ultralong CDRH3s (termed ulCABs in this work) have been solved to date [6,7,8,9,10,11,12]. Generally, CDRH3s of ulCABs exhibit a globular knob at the distal end of the stalk (Figure 1). These knobs contain three short antiparallel β-strands, which are connected by two hypervariable loops [6]. Knobs lack a distinct hydrophobic core, but are instead stabilized by several disulfide bonds [8]. They are connected to the globular immunoglobulin core domain by the so-called stalk, which consists of two antiparallel β-strands (Figure 1). In the crystal structures, the stalks generally protrude in an extended conformation from the core of the antibody, resulting in a similar knob–core distance. However, slight variations in the length, curvature, and tilt angle of the stalk affect the knob’s position relative to the core [5]. Overall, the crystal structures convey the picture of a rather rigid core–stalk–knob arrangement in an extended conformation.

However, the rigidity of this topology is questioned due to the presence of extensive crystal packing [11], variations in knob orientations among different copies within the asymmetric unit, or missing electron density for the knobs in some ulCABs [8]. Molecular dynamics (MD) simulations of the NC-Cow1 ulCAB revealed that the knob indeed undergoes significant motions relative to the immunoglobulin core domain [13]. Nevertheless, it remains unclear whether knob motions are a general feature of ulCABs and whether their magnitude is similar in various ulCABs.

To address this question, we have performed a large-scale comparative MD study including all 19 unique ulCAB structures available at the beginning of this study (21 April 2024). Our major aim was to characterize the type and magnitude of the knob domain motions. In addition, we identified hinge residues in the stalk region and investigated intramolecular interactions that stabilize alternative knob positions.

## 2. Methods

A BLAST 2.15 search using the bovine antibody SKD (PDB ID: 8EDF [10]) as a query sequence identified 18 structures of other nonredundant CABs with ultralong CDRH3s. To assess sequence conservation and to define domain boundaries, a structure-based multiple sequence alignment (MSA) of the heavy and light chains was performed using Promals3D [14]. Several of the selected ulCAB structures contained unresolved residues, particularly in flexible loop regions (Table 1). These missing segments were reconstructed using the ModLoop server [15] to ensure structural completeness.

All molecular dynamics (MD) simulations were carried out using AMBER 22 [16] with the ff14SB force field [17]. Systems were solvated in TIP3P water [18] within a truncated octahedral box, ensuring a 15 Å buffer between the protein and box edges to allow for large domain motions. Systems were neutralized using Na⁺ or Cl^−^ ions, respectively. A three-step minimization protocol was applied. In the first step, only water molecules were minimized while the solute was restrained. In the second step, backbone-restrained minimization was performed, allowing the side chains to relax. In the final step, full-system minimization was conducted with all restraints removed. Each step consisted of 2500 steps of steepest descent minimization followed by 2500 steps of conjugate gradient minimization. The systems were then equilibrated through a stepwise heating protocol. The temperature was raised from 10 K to 250 K over five heating steps of 0.2 ns each (totaling 1 ns), followed by a 0.5 ns heating phase to 300 K. Throughout these stages, backbone atoms were restrained using a harmonic potential of 5 kcal·mol^−1^·Å^−2^. Three independent production simulations (A, B, C) with different counter ion distributions were run for 1 μs at 300 K using a Berendsen thermostat and constant pressure (1 bar) with isotropic position scaling. All bonds involving hydrogen were constrained using the SHAKE algorithm [19], allowing a 2 fs time step. Particle mesh Ewald (PME) was used for long-range electrostatic interactions [20]. Minimization and equilibration were performed on CPUs, while production simulations were executed on NVIDIA A40 GPUs using pmemd.cuda [21,22,23].

Trajectory analysis was carried out using cpptraj [24], including calculations of root-mean-square deviation (RMSD), inter-residue distances, and linear interaction energies, while MM/GBSA binding free energies were calculated with MMPBSA.py [25,26]. In addition, global translational and rotational motions of the knob domain were evaluated using the latest cpptraj implementation [27]: first, the core domain was fitted on the initial structure, and then, in a second step, the knob domain was fitted accordingly, and the total translation and rotation required for the fitting were saved. This analysis allows for a detailed assessment of knob domain conformational sampling. In addition, backbone torsion-angle fluctuations (TAFs) were calculated as described in [28]. Plots were generated in Python 3.12.0 using Matplotlib 3.10.5 [29], and molecular visualizations were prepared using VMD [30], Rasmol [31], and PyMOL [32].

## 3. Results

### 3.1. Overall Dynamics and Knob Motions

**Figure 2:**
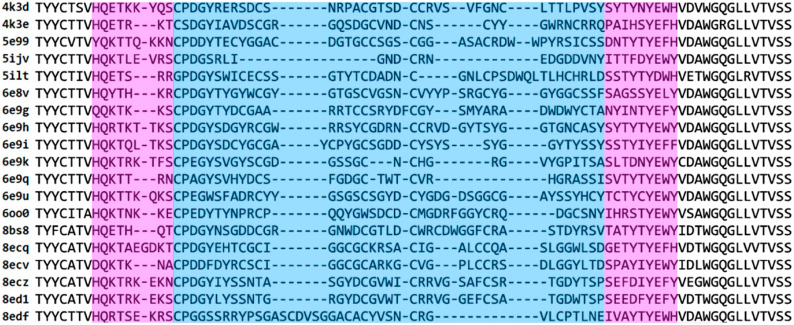
Multiple sequence alignment for the heavy chains of the 19 ulCABs. Since the core domains of the HCs are highly similar, the alignment is only shown for the segment containing the stalk (magenta) and knob (blue).

To study cow antibodies with ultralong CDRH3s (ulCABs), we collected all nonredundant ulCAB structures from the PDB as of 21 April 2024. For the resulting set of 19 ulCABs, a structure-based multiple sequence alignment reveals high sequence diversity for the knob domain and the adjacent stalk regions (Figure 2). To investigate how these differences affect ulCAB dynamics, 1 µs MD simulations were performed in triplicate. The systems were first analyzed with respect to their overall dynamics and conformational stability.

**Figure 3:**
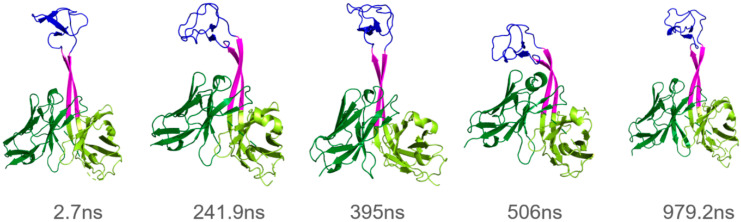
Representative snapshots from the 4k3d simulation (runA) illustrating the general type of knob motion. The time points, at which the conformations were observed, are given below the structures. Color coding is the same as in Figure 1.

For all systems, the RMSDs of the ulCAB core domain are low (range 1.11–1.92 Å), indicating that the globular parts of the HC and LC, as well as the interaction between these two domains, remain stable over the simulation time (Table 2). In contrast, the RMSDs for the overall systems are significantly higher (Table 2). A visual inspection of the ulCAB motions, exemplified for 4k3d in Figure 3, reveals that the higher RMSD mainly originates from the knob’s motions relative to the protein core. The range of overall RMSDs from 1.81 to 6.28 Å indicates that the extent of the knob motions differs between the systems investigated.

**Figure 4:**
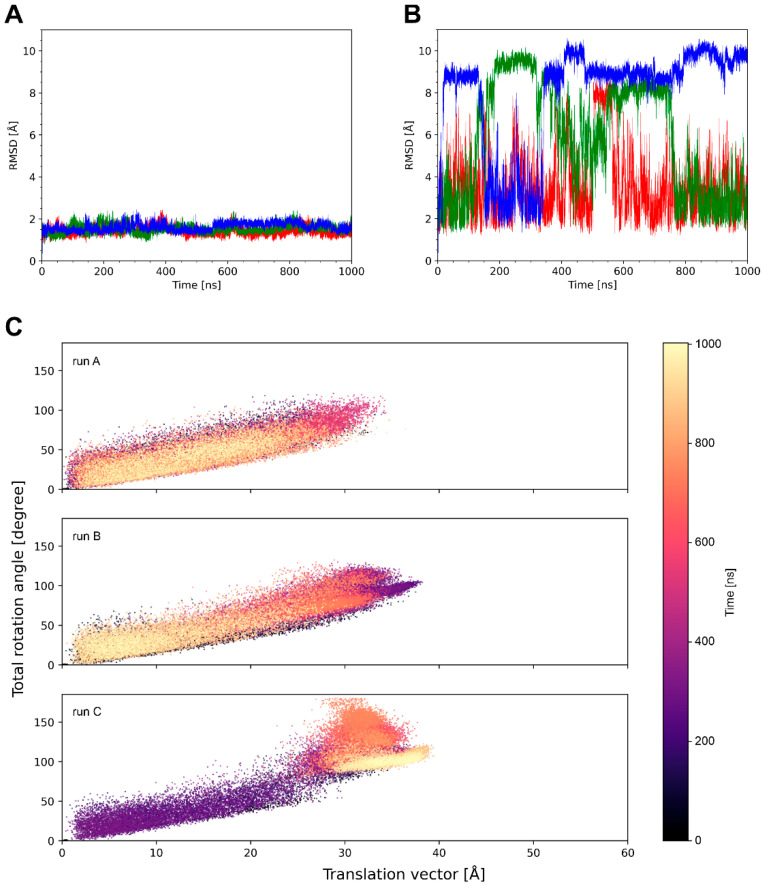
A summary of the simulation data for system 4k3d. RMSD of the (**A**) ulCAB core and (**B**) the entire ulCAB system over the simulation time. Data for runA, runB, and runC are shown in red, green, and blue, respectively. (**C**) Translational and rotational changes in the knob position with respect to the crystal structure observed over the simulation time.

**Figure 5:**
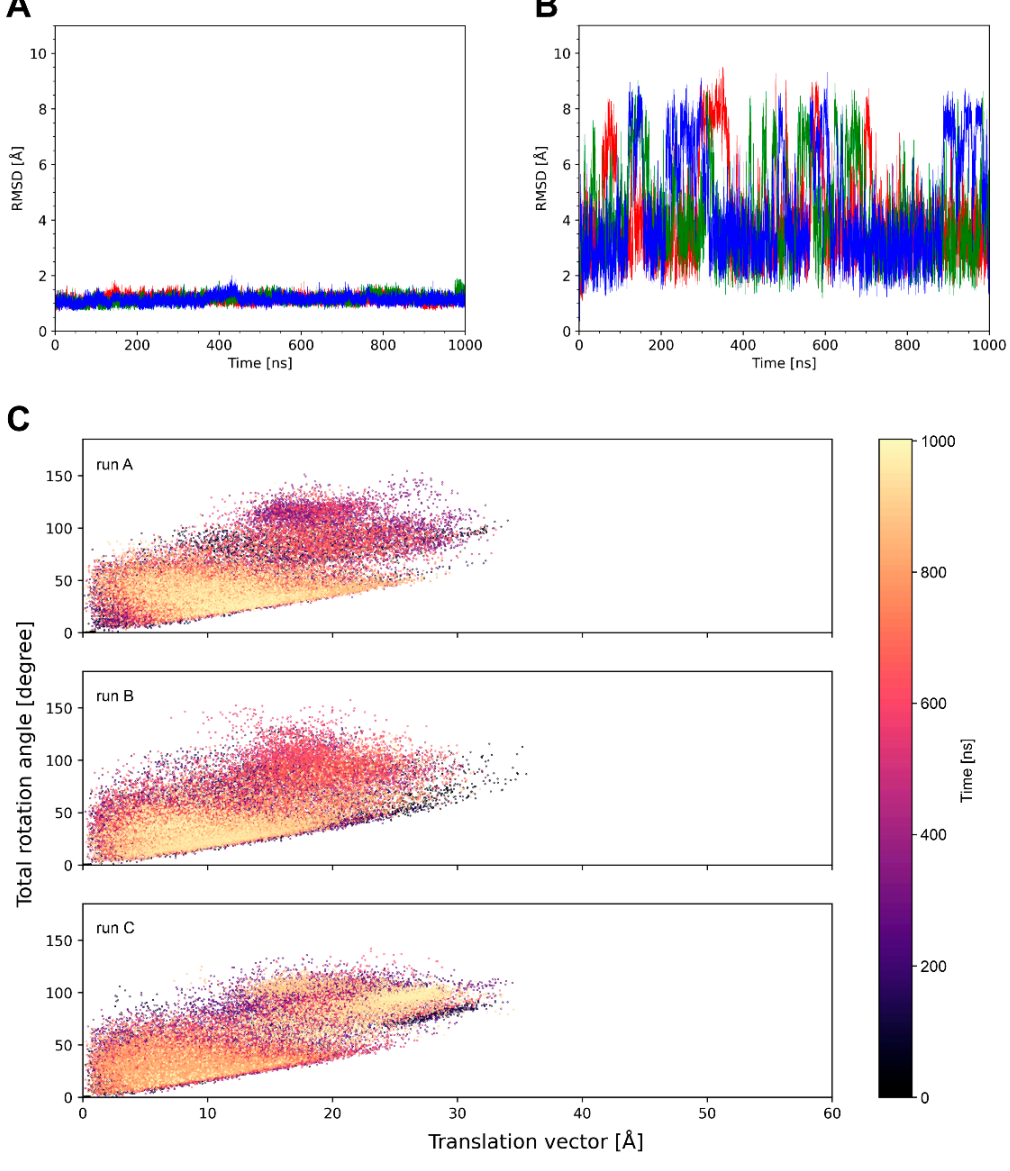
A summary of the simulation data for system 8edf. RMSD of the (**A**) ulCAB core and (**B**) the entire ulCAB system over the simulation time. Data for runA, runB, and runC are shown in red, green, and blue, respectively. (**C**) Translational and rotational changes in the knob position with respect to the crystal structure observed over the simulation time.

**Figure 6:**
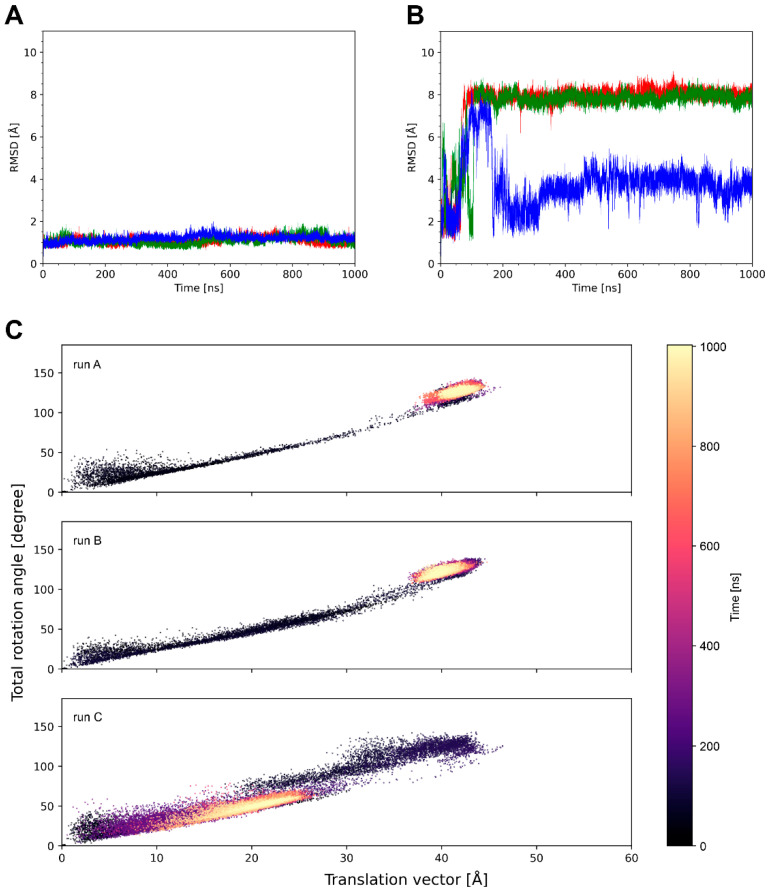
A summary of the simulation data for system 5ilt. RMSD of the (**A**) ulCAB core and (**B**) the entire ulCAB system over the simulation time. Data for runA, runB, and runC are shown in red, green, and blue, respectively. (**C**) Translational and rotational changes in the knob position with respect to the crystal structure observed over the simulation time.

The type of knob motion was further assessed by calculating the translational and rotational (tra/rot) offset of the knob over the simulation time (Table 2). With the exception of 6e9u, where the knob is covalently linked by disulfide bonds to the stalk, all systems exhibit a translational offset ranging from 9 to 32 Å. This indicates significant knob dynamics and suggests that the magnitude of these movements is system-dependent. The range of RMSD values and tra/rot offset is rather continuous (Table 2), rendering it difficult to divide the set of 19 ulCABs into distinct classes based on their knob dynamics. A closer inspection of the individual systems also revealed that the timescale and reversibility of the knob motions differ significantly. This is exemplified for three representative systems in Figure 4, Figure 5 and Figure 6 and Supplementary Videos S1–S3.

System 4k3d shows a stable ulCAB core (Figure 4A) and significant dynamics of the knob (Figure 3 and Figure 4B). Bent knob conformations (as evidenced by high RMSD values in Figure 4B) exist for time periods of >100 ns and reversibly exchange with extended conformations that closely resemble the conformation observed in the crystal structure (Figure 3). However, there are some differences between the three simulation runs with respect to the overall sampling of the knob conformations (Figure 4B and Figure S1). This observation can be explained by the fact that large-scale domain motions, like knob motions, frequently occur on µs to ms timescales [33,34] and are therefore not fully covered by conventional MD simulations. Despite the differences between the individual simulation runs, the tra/rot plots of 4k3d suggest that the knob exhibits a similar conformational freedom in all three simulation runs (Figure 4C).

The properties of system 8edf are shown in Figure 5. The tra/rot plot (Figure 5C) shows that the translational and rotational degrees of freedom are similar to those detected in 4k3d (Figure 4C). However, compared to 4k3d, the motions in 8edf are relatively fast, and conformations that deviate by more than 6 Å RMSD from the starting structure only exist for shorter (<50 ns) periods of the simulation time (Figure 5B).

Unlike the systems analyzed above, which formed distinct knob conformations only reversible over certain periods of the simulation time, 5ilt adopts knob conformations that deviate significantly from the crystal structure and are maintained over the entire simulation time (Figure 6B). The tra/rot plots (Figure 6C) show that the system quickly deviates from the conformation observed in the crystal structure and undergoes large translational shifts in the knob of up to 40 Å. This state represents a minimum in runs A and B and remains stable until the end of the simulation. In runC, a distinct state with a tra/rot offset of ~20 Å/50 deg is formed (Figure 6C).

### 3.2. The Dynamics of the Stalk

**Figure 7:**
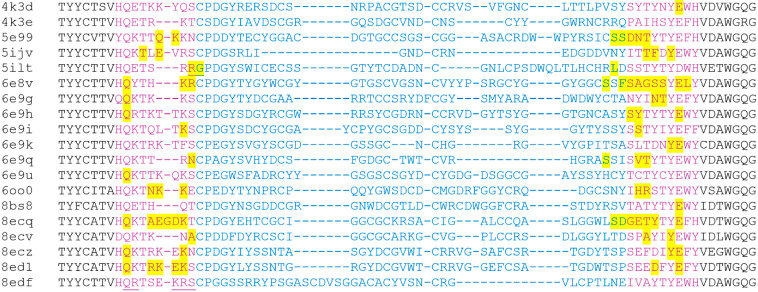
Multiple sequence alignment indicating the residues with large torsion-angle fluctuations. Residues of the stalk and knob are colored in purple and blue, respectively. Residues with backbone torsion-angle fluctuations > 0.6 are highlighted in yellow. The analysis is restricted to the stalk and the two adjacent residues of the knob. Particular sites discussed in the text are underlined.

Visual inspection of the ulCAB simulations shows that motions of the knob detected in the present study result from bending motions in the stalk region (exemplarily shown for 4k3d, 8edf, and 5ilt as Supplementary Videos S1–S3). This observation confirms the previous notion, derived from the analysis of crystal structures [8] and an explicit MD simulation of 6oo0 [13], that the stalk represents the major site of flexibility. To gain further insight, we investigated whether bending occurs at the same site of the stalk in different systems. For that purpose, we analyzed the backbone torsion-angle fluctuations (TAFs) for the stalk and the two knob residues adjacent to the stalk. Residues that exhibit large fluctuations (>0.6) of their φ or ψ angles are highlighted in Figure 7. The data shows that the site and number of residues with large TAFs significantly differs between the systems investigated. We found no clear correlation between the number of residues with large TAFs (Figure 7) and the magnitude of the knob displacement observed (Table 2).

**Figure 8:**
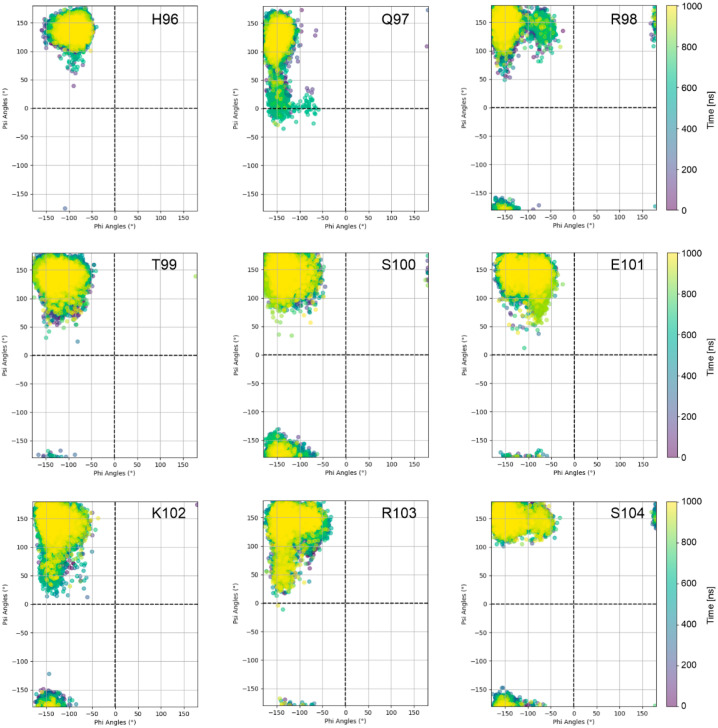
Ramachandran plots indicating the structural changes in the N-terminal strand of the stalk of system 8edf (runA). A separate Ramachandran plot is shown for each residue, and the phi/psi combinations are colored according to the simulation time.

Moreover, individual sequence properties of the stalk and adjacent knob residues may critically affect knob dynamics. For example, the particularly large knob displacement observed for 5ilt (Figure 6; Table 2) is likely due to the presence of glycine at the N-terminus of the knob; at this sequence position, a cysteine is present in all other ulCABs investigated (Figure 7). However, residues with large TAFs are also observed in the stalk of systems like 6e9q or 6oo0 that exhibit a rather small overall translational knob offset (Table 2). In contrast, some systems like 8edf do not exhibit residues with large TAFs at all (Figure 7). A closer inspection of the N-terminal strand of the 8edf stalk reveals that the motions observed in this system (Figure 5; Supplementary Video S2) result from rather small changes in the φ and ψ angles either for residues Q97/R98 or for the stretch K102-S104 (Figure 8).

### 3.3. Interactions Between the Knob and the Light Chain

From a visual inspection of the motions, we noted that the formation of bent conformations frequently coincided with the presence of interactions between the knob and core domain (see Figure 3 and Supplementary Videos S1–S3). We therefore quantified these interactions by calculating the van der Waals (vdW) interaction energy between the knob and the HC or LC part of the core domain. The vdW interaction energy was selected as the readout because, due to its short-range nature, favorable interaction energies reflect direct contacts. The analysis shows that most of these interactions are formed with the LC and only a few with the HC core domain (Table 3 and Table S3).

**Figure 9:**
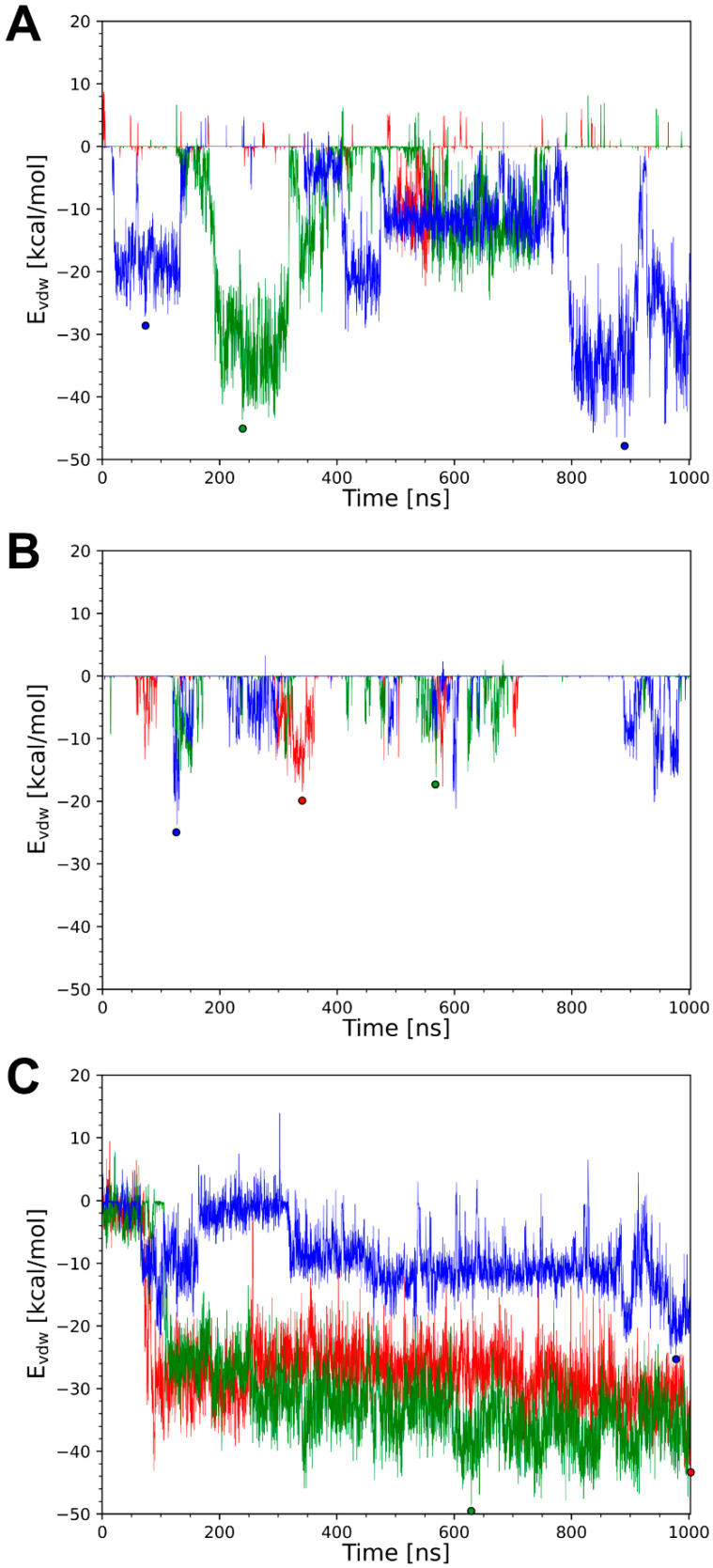
Plots of the van der Waals interaction energy between knob and LC for (**A**) 4k3d, (**B**) 8edf, and (**C**) 5ilt. Data for runA, runB, and runC are shown in red, green, and blue, respectively. Dots denote local energy minima that were investigated in greater detail.

A more detailed analysis for systems 4k3d, 8edf, and 5ilt reveals that there is almost quantitative correlation between the formation of bent conformations and the presence of favorable knob–LC interactions. For 4k3d, the time course of knob–LC interaction energy is explicitly shown in Figure 9A. The data shows that intervals with tight vdW interactions, e.g., the time interval from 200 to 300 ns for runB (Figure 9A; green), coincide with the presence of a bent conformation as indicated by large RMS deviations from the crystal structure (Figure 4B).

In 8edf, the knob–LC interaction is weaker than in 4k3d (Figure 9B; Table 3). This is in line with the observation that conformations, which significantly deviate from the crystal structure, are only formed for shorter periods of time (Figure 5B). Nevertheless, also for 8edf, favorable knob–LC interactions correlate with conformations deviating from the extended crystal structure (see, for example, runA at ~300 ns in Figure 5B and Figure 9B).

Compared to 8edf, the knob–LC interaction is significantly stronger in 5ilt (Figure 9C; Table 3) and correlates with the formation of stable knob conformations that are clearly distinct from the crystal structure (Figure 6B,C and Supplementary Video S3). Interestingly, there are large differences in the tra/rot offset (Figure 6C) and the knob–LC interaction energy (Figure 9C) between runC and the other two simulation runs, which prompted us to investigate the knob–LC interactions in more detail. For that purpose, snapshots representing the local energy minima of the knob–LC interaction were selected for further analysis.

**Figure 10:**
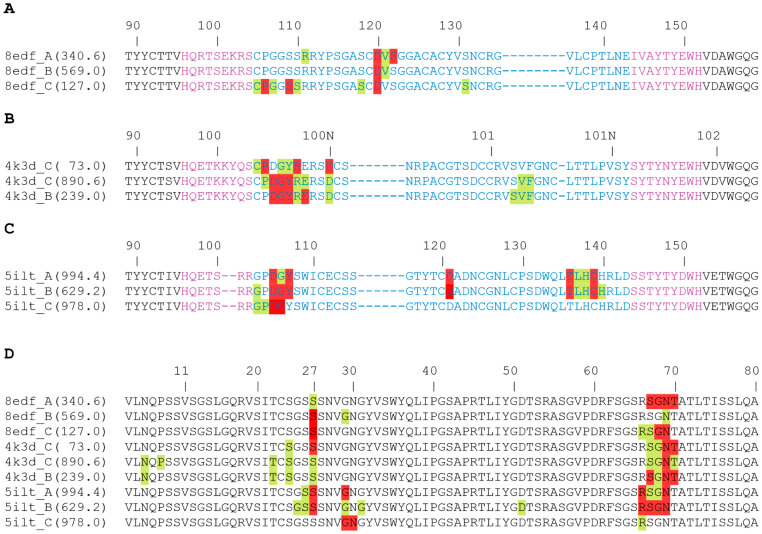
Residues involved in knob–LC interactions. (**A**) Sequence of the 8edf knob region indicating the residues that interact with the LC. Three representative snapshots that represent local energy minima are listed. For each snapshot, the simulation run and the respective time point in the simulation (in ns; in brackets) are specified. Residues of the stalk and knob are colored in purple and blue, respectively. Residues forming only nonpolar interaction are marked in green; residues additionally forming polar interactions are marked in red. Sequence numbering according to the PDB entry. (**B**) Sequence of the 4k3d knob region indicating the residues that interact with the LC. Please note that according to the 4k3d PDB entry, the Kabat numbering scheme is used here. Color coding is the same as in (**A**). (**C**) Sequence of the 5ilt knob region indicating the residues that interact with the LC. (**D**) Sequence of the LC region that forms interactions with the knob in 8edf, 4k3d, and 5ilt. Since the sequence of this region is identical in all three ulCABs, only one single sequence alignment is shown here.

**Figure 11:**
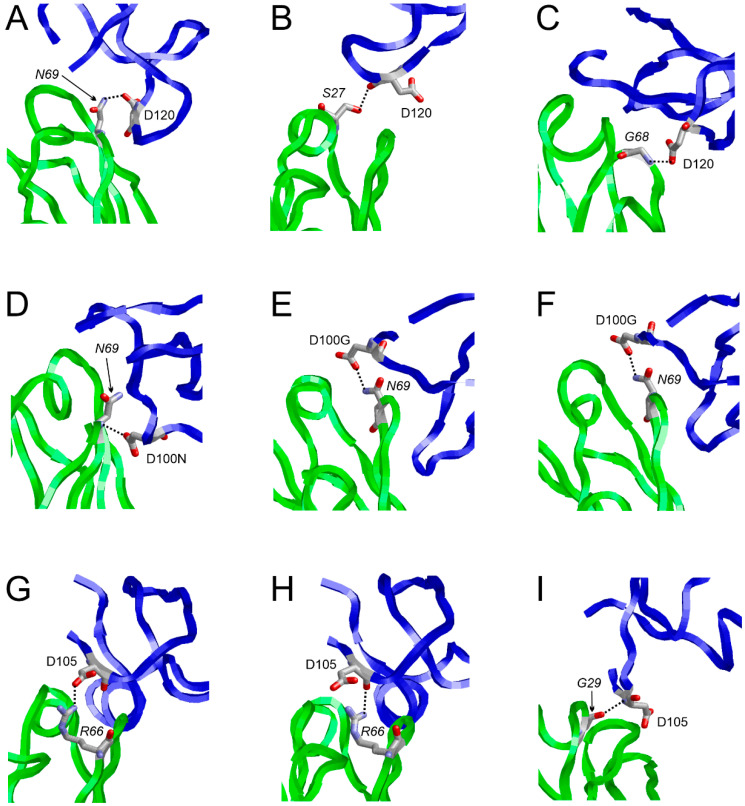
Representative knob–LC interactions observed for structures at local energy minima. The knob and LC are shown in blue and green, respectively. Selected interacting residues are shown in stick presentation and are labeled (labels in italics denote residues of the LC). Polar interactions are denoted by dotted lines. The following structures are depicted: (**A**) 8edf, runA, 340.6 ns; (**B**) 8edf, runB, 569.0 ns; (**C**) 8edf, runC, 127.0 ns; (**D**) 4k3d, runC, 73.0 ns; (**E**) 4k3d, runC, 890.6 ns; (**F**) 4k3d, runB, 239.0 ns; (**G**) 5ilt, runA, 994.4 ns; (**H**) 5ilt, runB, 629.2 ns; (**I**) 5ilt, runC, 978.0 ns.

For 8edf, such local energy minima were observed at 340.6 ns in runA, 560.0 ns in runB, and 127.0 ns in runC (Figure 9B). For these structures, the knob–LC interactions were investigated in detail, as shown in Figure 10 and Figure 11. In all three snapshots selected, the heavy chain residue D120_H_ forms interactions with the light chain (Figure 10A); however, the interacting residue of the LC is different in the three simulation runs: in the selected snapshots, the main interaction partners of D120_H_ are N69_L_, S27_L_, and G68_L_ in runs A, B, and C, respectively (Figure 11A–C).

For system 4k3d, the main interacting surface patches are P100F_H_-D100N_H_ of the knob and S67_L_-T70_L_ of the LC in all snapshots analyzed (Figure 10B,D). However, as already observed for 8edf, the structural details of the interactions may differ. This is exemplified by the interactions formed by N69_L_: in runC (73 ns), N69_L_ interacts with D100N_H_, whereas in runB (239 ns) and runC (890.6 ns), N69_L_ interacts with D100G_H_ (Figure 11D–F). Thus, for 4k3d, different knob–LC interactions are sampled within the same simulations, but the same pattern of interactions also can be found in two different simulations.

The latter observation is also supported by runs A and B of the 5ilt simulations. In runA (994.4 ns) and runB (629.2 ns), a similar interaction is formed between D105_H_ and R66_L_ (Figure 11G,H). This interaction is enforced by additional polar interactions formed by residues D121_H_ and T136_H_ with the LC in both simulation runs. In contrast, in runC (978 ns), D105_H_ interacts with G29_L_ instead of R66_L_ (Figure 11I). This offers an explanation for the different knob position compared to runs A and B (Figure 6C).

In summary, the present work discovered attractive knob–LC interactions as a widespread structural feature present in about half of the systems studied (Table 3). The molecular details of the knob–LC interaction differ between the systems and may even differ between the simulation runs of the same system (Figure 10 and Figure 11).

The HCs of ulCABs preferentially pair only with a limited set of highly conserved LCs and the structures of ulCABs in complexes with antigens have shown that the LCs do not contribute to the antigen-binding site [9,10]. LCs of ulCABs are highly similar in sequence and were shown to confer a unique combination of favorable physicochemical properties including efficient secretion from mammalian cells, strong dimerization, high stability, and resistance to aggregation [35]. Our study suggests that ulCAB LCs may have a further favorable property, namely the ability to stabilize knob domains. A role of the LC for the stabilization of long CDRH3s has already been reported for several nonbovine antibodies [36].

However, it is not yet clear whether the LC-interacting knob conformations found in the present study are more favorable for antigen binding than the extended conformations found in the static crystal structures. This point requires further experimental investigation, e.g., by the design of LC mutants that disrupt knob–LC interactions. This might be carried out by inserting charged residues in the LC that cause electrostatic repulsion with the knob domain. A complementary approach to the disruption of knob–LC interactions would be the design of variants that enhance knob–LC interactions. Such an approach may be inspired by the structure of system 6e9u, which exhibits two disulfide bonds between the knob and stalk that restrict the motion of the knob (Table 2) and fix the knob in an LC-interacting position (Table 3).

## 4. Discussion

### 4.1. Knobs Are Generally Highly Dynamic

One of the key findings of this study is that the position of the knob in ulCABs relative to the globular core domain tends to be highly dynamic (Table 2). The only exception is system 6e9u, where the knob is covalently linked to the stalk by disulfide bonds. In all other systems, the average knob position deviates by 9–32 Å from that observed in the crystal structure (Table 2), indicating that the extended conformation observed in the crystal structure may not represent a deep energy minimum in solution and that there are additional energetically favorable knob orientations.

This observation does not contradict the experimental structures but can mainly be explained by extensive crystal packing. In their study, Dong et al. found that all knobs with traceable electron density form crystal contacts with other structural elements in the protein crystals, restricting their motions and making them visible [8]. Crystal packing was also suggested by Clarke et al. to have a significant effect on the exact positioning of ultralong CDR3H loops and on the knob orientation of ulCAB D08 (PDB-ID: 8bs8) [11]. This observation is also supported by a comparison of the individual structures that form the asymmetric unit in the crystal. For the antibodies BOV-1 (PDB-ID: 6e8v) and BOV-4 (PDB-ID: 6e9i), the stalks of different copies within one asymmetric unit exhibit structural variations, demonstrating that the ul-CDRH3 stalks can perform tilting and twisting motions due to their inherent flexibility [8].

Evidence for the presence of a flexible hinge between the knob and stalk was also found in a cryo-EM structure of the Fab NC-Cow1 (PDB-ID: 6oo0) in a complex with the fully glycosylated HIV envelope trimer BG505 SOSIP.664 (PDB-ID: 6pw6). The cryo-EM map showed well-resolved density for the NC-Cow1 knob domain but poorly resolved density for the rest of the Fab structure, indicating flexibility in the region between the knob and the core domain of NC-Cow1 [9]. This flexibility was further confirmed by MD simulations of NC-Cow1 [13], which to the best of our knowledge, represent the only explicit MD simulation of a ulCAB to date. For a more comprehensive investigation of ulCAB dynamics, the present comparative MD study included 19 unique ulCAB structures. The results of our study reveal that pronounced knob dynamics is a general feature of ulCABs rather than an exception.

### 4.2. The Magnitude of Knob Motions Is System-Dependent

Although 18 of the 19 ulCABs investigated exhibit significant knob dynamics as a common feature, a quantitative comparison of the dynamic properties of these 18 systems revealed remarkable differences:

(i) The average translational offset of the knobs ranges from 9 to 32 Å (Table 2).
(ii) Alternative conformations may be formed transiently or permanently over the simulation time of 1 µs (Figure 4, Figure 5 and Figure 6).
(iii) The frequency of the knob motions differs significantly (Figure 4B, Figure 5B and Figure S1; Supplementary Videos S1–S3).
(iv) About half of the systems show energetically favorable knob–LC interactions over the simulation time with significant differences in the interaction energies (Table 3).
(v) The sequence position and number of stalk residues with large torsion-angle fluctuations, which contribute to knob bending, differ significantly (Figure 7).

The above analyses show that knob motions are system-dependent and cover a continuous range of dynamic properties. No clear correlation was found between knob motions and either knob sequence or the stalk length. Therefore, the diverse dynamics rather reflects the high ulCAB sequence diversity [4] and prevents the definition of distinct groups of ulCABs based on the dynamic properties of their knobs. This is an important finding as it demonstrates that there are no simple sequence features that can be used to predict the type or magnitude of knob motions. In this context, MD simulations can help to gain a more detailed insight into knob motions of ulCABs.

### 4.3. Implications of Knob Dynamics for Biological Function and Antibody Design

The observation of large knob motions in most of the systems investigated raises the question about possible beneficial effects for ulCAB function. Dong et al. proposed that the thermodynamics and/or kinetics of antibody binding to the corresponding antigens may be modulated by flexible stalks and hinges [8]. Dong et al. also speculated that it may be beneficial from a kinetic perspective for ulCABs to have flexible stalks and stalk–knob junctions in order to recognize epitopes in deep cavities or crevices that otherwise could not be recognized [8].

Svilenov et al. [13] performed a detailed mutational analysis of the NC-Cow1 ulCAB, in which the stalk was either deleted, replaced by glycines, or extended by 5 or 10 residues. Compared to the wildtype, all constructs exhibited reduced thermal stability and reduced binding affinity for the HIV-1 Env antigen. In addition, extending the stalk also reduced the secretion levels of the corresponding Fab fragments from mammalian cells [13]. Interestingly, the reduced binding affinity of the mutated constructs mainly resulted from a reduced on-rate, which supports the idea that the length and flexibility of the stalk is optimized to enable kinetically favorable knob–antigen recognition. In this context, the knob–LC interactions found in the present study could contribute to fine-tuning the knob dynamics, thereby optimizing the kinetics of antigen recognition. However, further research is needed to confirm this.

Understanding stalk–knob dynamics could also help to optimize the design of isolated knob constructs. Knobs are potent mini-antibodies [37], but the exact length of the termini is crucial for their stability and antigen-binding properties [10,38]. When creating a mini-antibody based on the ulCAB 2G3 knob, Huang et al. [10] observed a loss of activity due to the removal of the stalk, which clamps the N- and C-termini of the knob. To improve knob stability and activity, the authors optimized two amino acids at each of the N- and C-termini of the knobs using phage display. Two sequences were identified that showed eight-fold pseudovirus neutralization potency enhancement compared to the original knob construct [10]. As an alternative, the stability of isolated knobs may also be improved by replacing stalk regions with stable structural elements, like coiled-coil domains [38]. In this context, molecular dynamics (MD) simulations of ulCABs could help identify suitable knob fragments by suggesting appropriate truncation points based on flexible sites in the stalks. Furthermore, MD simulations could be used to study the conformational stability of isolated knobs and propose ways to enhance their stability.

## Figures and Tables

**Table 1:** An overview of the simulated systems, including the length of the constructs investigated. PDB-ID is used in this paper as an abbreviation for the systems.

No.	System (PDB-ID)	Light Chain	Heavy Chain	Protein Residues	Residues Modeled	Water Molecules	Total Atoms
1	4k3d [7]	V3-G107	V2-S113	283	-	29,230	91,722
2	4k3e [7]	V3-G107	V2-S113	273	3	26,599	83,732
3	5e99 [6]	V3-G107	V2-S168	287	-	25,759	81,274
4	5ijv [6]	V3-G107	V2-S149	271	-	27,250	85,539
5	5ilt [6]	V3-G107	V2-S166	288	-	24,564	77,732
6	6e8v [8]	V3-G112	V28-S195	284	-	24,491	77,426
7	6e9g [8]	V3-G111	L4-S167	279	10	26,918	84,737
8	6e9h [8]	V3-G112	Q29-S197	282	7	28,469	89,436
9	6e9i [8]	V3-L111	V28-S194	283	-	30,014	93,998
10	6e9k [8]	V3-G112	Q3-S162	275	5	22,086	70,105
11	6e9q [8]	V3-L111	V2-S158	271	18	21,871	69,458
12	6e9u [8]	V3-G112	V2-S171	288	-	26,203	82,603
13	6oo0 [9]	E3-G107	V2-S165	283	4	27,374	86,178
14	8bs8 [11]	V31-G139	V30-S197	288	1	27,379	86,142
15	8ecq [10]	V3-G107	V2-S166	285	-	22,681	71,984
16	8ecv [10]	V3-G107	V2-S174	280	-	22,415	71,212
17	8ecz [10]	V3-G107	V2-S176	286	11	29,643	92,975
18	8ed1 [10]	V3-G107	V2-S166	286	-	28,656	90,015
19	8edf [10]	V3-G107	V2-S166	281	-	29,327	91,962

**Table 2:** Conformational stability of the 19 systems. The values indicate the deviation from the conformation found in the crystal structure. All values are averaged over the entire simulation time and the three simulation runs. Values for the individual simulation runs are listed in Supplementary Tables S1 and S2.

System	RMSD [Å] (Core Domain)	RMSD [Å] (Entire ulCAB)	Translational Offset [Å] (Knob Domain)	Rotational Offset [deg] (Knob Domain)
4k3d	1.52	5.76	21.36	69.11
4k3e	1.15	3.11	15.18	37.20
5e99	1.24	5.91	25.23	67.94
5ijv	1.50	3.68	14.35	43.73
5ilt	1.11	6.25	32.58	95.71
6e8v	1.41	5.66	22.35	79.28
6e9g	1.92	6.28	20.13	70.66
6e9h	1.40	6.27	26.97	93.45
6e9i	1.43	3.42	10.82	28.74
6e9k	1.36	3.62	10.85	37.01
6e9q	1.35	3.30	10.79	48.31
6e9u	1.44	1.81	3.10	13.53
6oo0	1.38	2.92	9.05	33.37
8bs8	1.40	4.36	14.36	43.12
8ecq	1.44	4.66	19.99	65.15
8ecv	1.33	4.02	15.94	62.00
8ecz	1.18	5.23	23.25	59.69
8ed1	1.31	4.78	15.69	54.05
8edf	1.14	4.20	12.03	48.25

**Table 3:** Van der Waals interaction energy (E_vdW_ in kcal/mol) between the knob and light chain for the 19 systems investigated. Values are averaged over the entire simulation time but shown separately for the three simulations runs. The last column gives the average value over all three simulation runs.

System	E_vdW_ (runA)	E_vdW_ (runB)	E_vdW_ (runC)	Average E_vdW_
4k3d	−0.59	−7.39	−13.28	−7.08
4k3e	−0.09	−0.59	−3.00	−1.23
5e99	−0.49	−2.15	−16.61	−6.41
5ijv	1.27	7.25	4.96	4.49
5ilt	−25.88	−29.66	−8.82	−21.45
6e8v	−6.88	−4.75	−6.76	−6.13
6e9g	−11.56	−10.84	−2.28	−8.22
6e9h	−4.32	−2.56	−10.77	−5.88
6e9i	−2.71	−3.81	0.17	−2.12
6e9k	−4.16	−11.43	−3.26	−6.28
6e9q	−0.10	−0.05	−0.24	−0.13
6e9u	−12.11	−13.75	−11.83	−12.56
6oo0	0.13	0.09	−0.03	0.06
8bs8	0.20	−0.43	−0.46	−0.23
8ecq	−5.91	−5.53	−4.23	−5.22
8ecv	−0.85	−0.51	−2.88	−1.41
8ecz	−0.92	−7.63	−0.63	−3.06
8ed1	−0.47	−1.30	−0.48	−0.75
8edf	−0.81	−0.84	−1.44	−1.03

## Data Availability

The original contributions presented in this study are included in the article/Supplementary Materials. Further inquiries can be directed to the corresponding author.

## Supplementary Materials

The following supporting information can be downloaded at https://www.mdpi.com/article/10.3390/antib14030070/s1. Figure S1: Summary of simulation data for all 19 systems investigated; Table S1: RMSD values for all 19 systems investigated; Table S2: Translational and rotational offset of knob domain position for individual simulation runs; Table S3: Van der Waals interaction energy between knob and core domain of heavy chain; Video S1: Dynamics of system 4k3d (runB); Video S2: Dynamics of system 8edf (runA); Video S3: Dynamics of system 5ilt (runA).
