# Supplementary material for: Comparative Molecular Dynamics Study of 19 Bovine Antibodies with Ultralong CDR H3
Source: Antibodies (Basel). 2025 Aug 13;14(3):70. doi: 10.3390/antib14030070 (PMC12371982; doi:10.3390/antib14030070)
Supplement: Supplementary file 1 [file antibodies-14-00070-s001.zip › Supplementary_Figure.pdf]

Supplementary Figure

Comparative molecular dynamics study of 19 bovine antibodies with ultralong CDR H3

Olena Denysenko [1], Anselm H. C. Horn [1,2], Heinrich Sticht [1,2*]

[1] Bioinformatics, Institute of Biochemistry, Friedrich-Alexander-Universität Erlangen-Nürnberg (FAU), Germany

[2] Erlangen National High Performance Computing Center (NHR@FAU), Friedrich-Alexander-Universität Erlangen-Nürnberg (FAU), Germany

* Correspondence: heinrich.sticht@fau.de

The following pages show Figure S1

Figure S1: Summary of the simulation data for all 19 systems investigated. For each system two RMSD plots are shown: The left panel shows the RMSD of the ulCAB core and the right panel shows the RMSD for the entire ulCAB system over the simulation time. The name of the system is indicated above the diagrams. Data for runA, runB, and runC is shown in red, green, and blue, respectively.

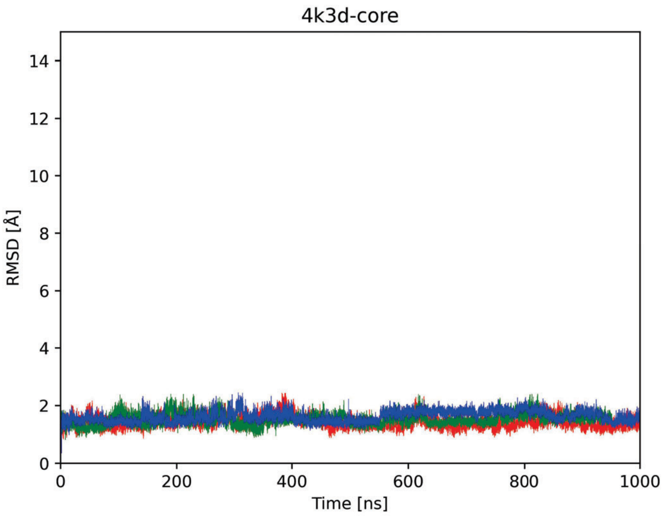
4k3d-core
RMSD [Å]
Time [ns]

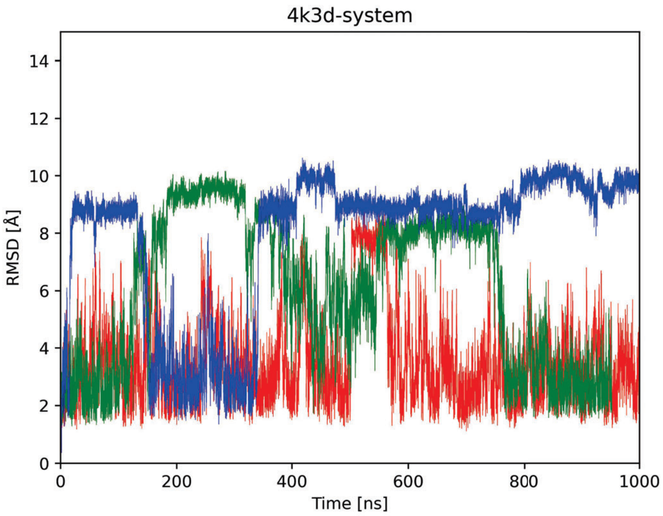
4k3d-system
RMSD [Å]
Time [ns]

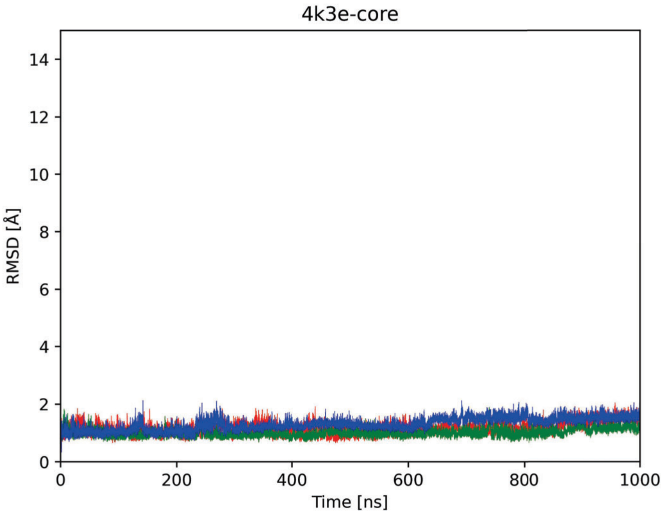
4k3e-core
RMSD [Å]
Time [ns]

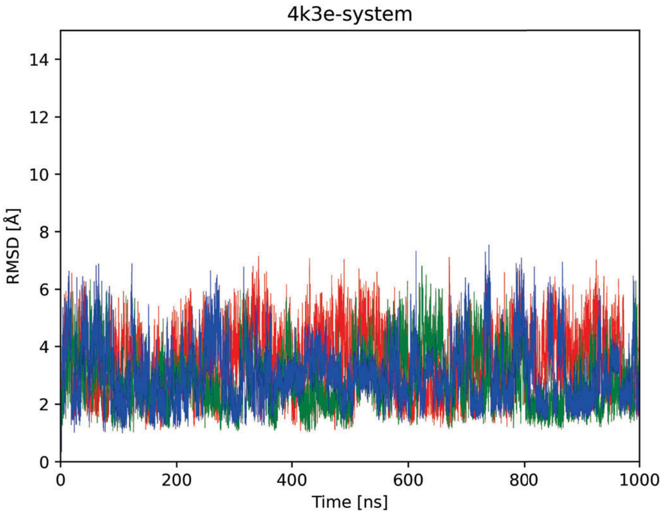
4k3e-system
RMSD [Å]
Time [ns]

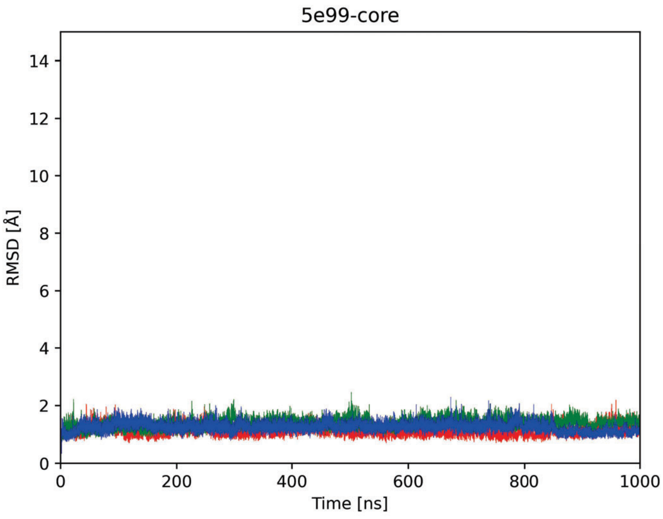
5e99-core
RMSD [Å]
Time [ns]
0
2
4
6
8
10
12
14
0
200
400
600
800
1000

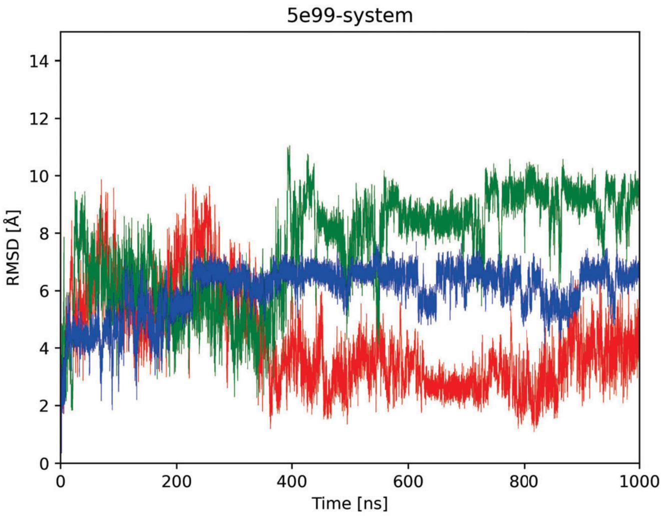
5e99-system
RMSD [Å]
Time [ns]
0
2
4
6
8
10
12
14
0
200
400
600
800
1000

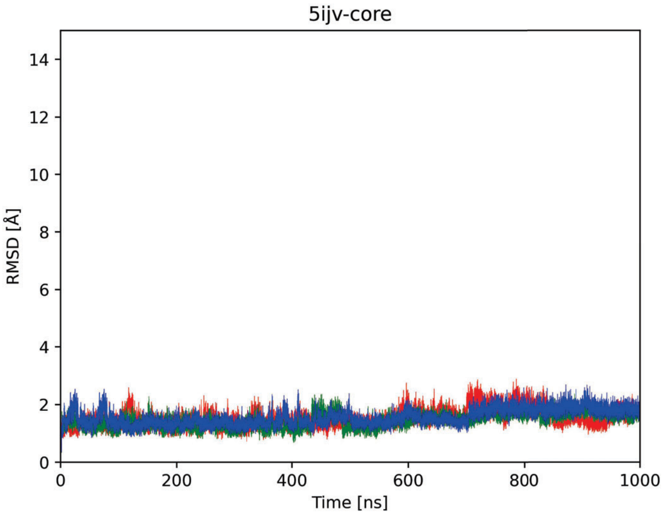
5ijv-core
RMSD [Å]
Time [ns]
0
2
4
6
8
10
12
14
0
200
400
600
800
1000

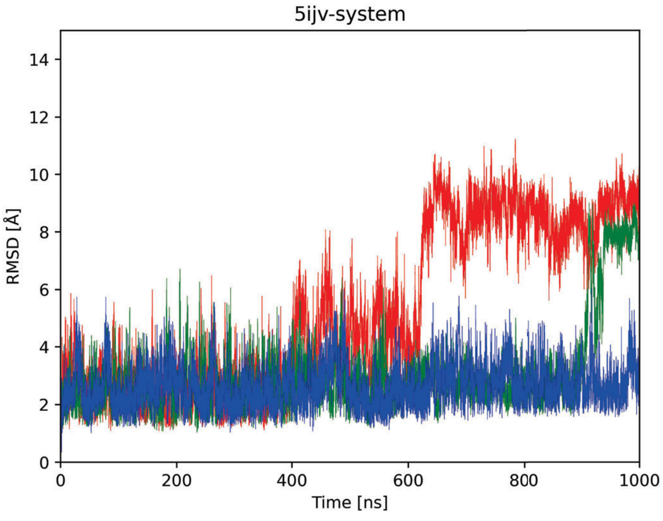
5ijv-system
RMSD [Å]
Time [ns]
0
2
4
6
8
10
12
14
0
200
400
600
800
1000

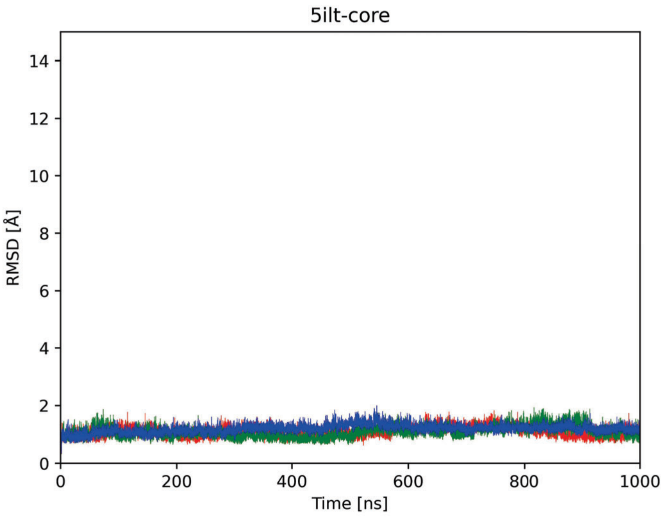
5ilt-core
RMSD [Å]
Time [ns]

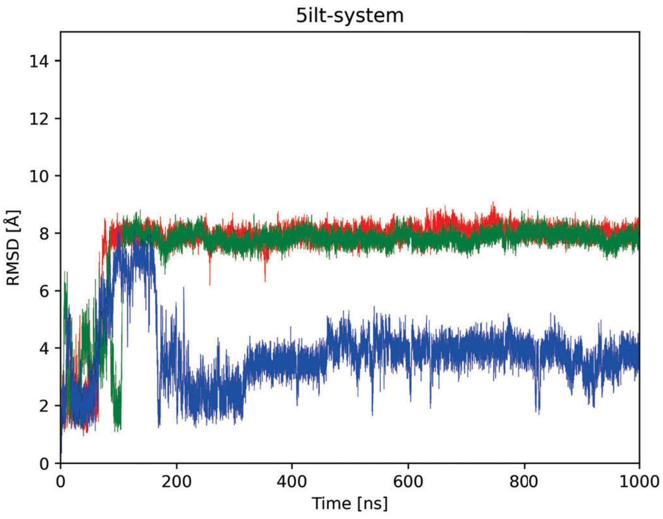
5ilt-system
RMSD [Å]
Time [ns]

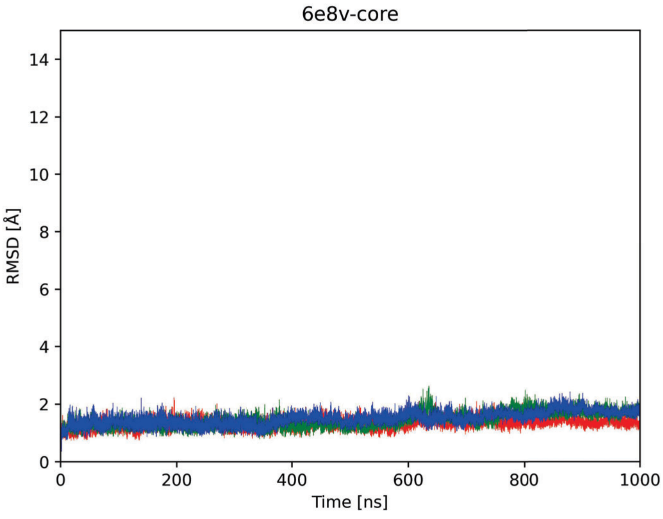
6e8v-core
RMSD [Å]
Time [ns]

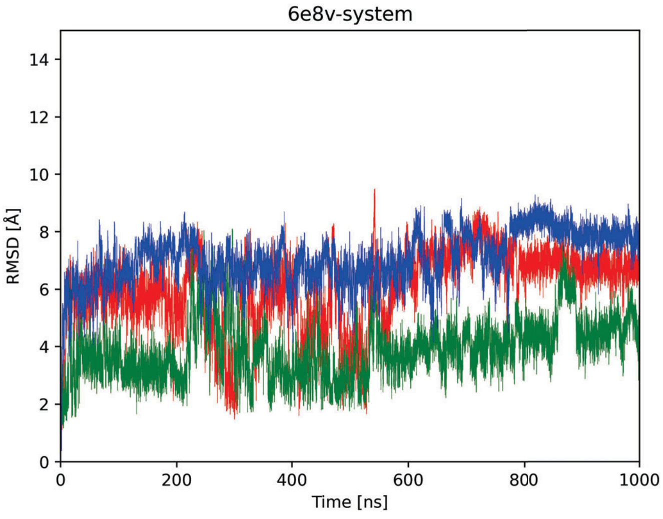
6e8v-system
RMSD [Å]
Time [ns]

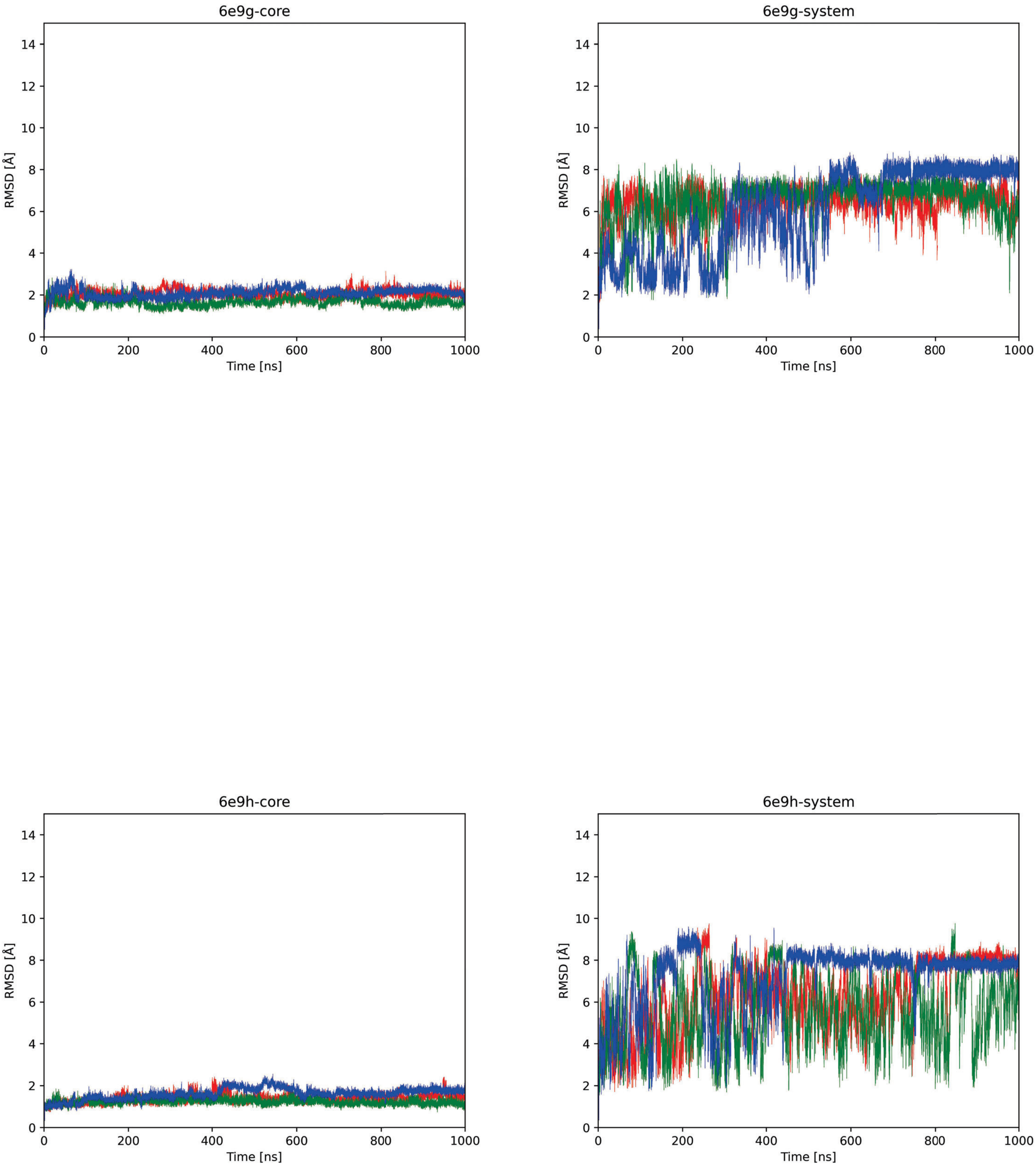
6e9g-core
6e9g-system
6e9h-core
6e9h-system
RMSD [Å]
Time [ns]
0
2
4
6
8
10
12
14
200
400
600
800
1000

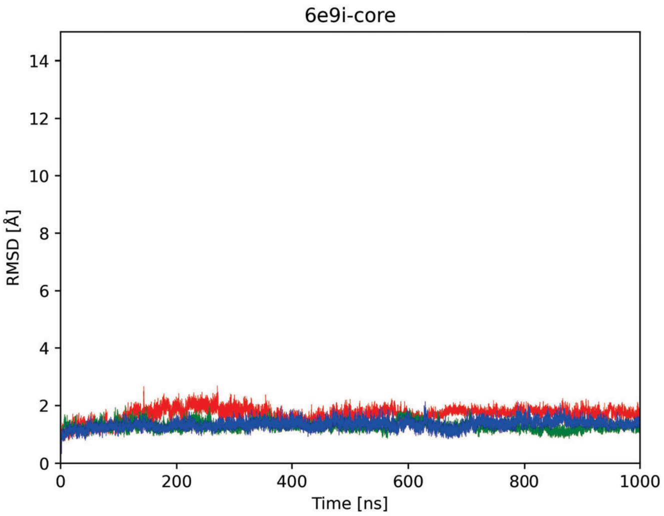
6e9i-core
RMSD [Å]
14
12
10
8
6
4
2
0
200
400
600
800
1000
Time [ns]

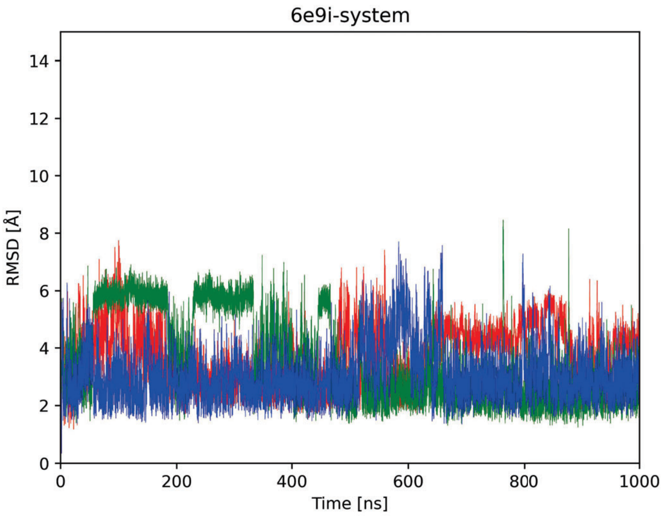
6e9i-system
RMSD [Å]
14
12
10
8
6
4
2
0
200
400
600
800
1000
Time [ns]

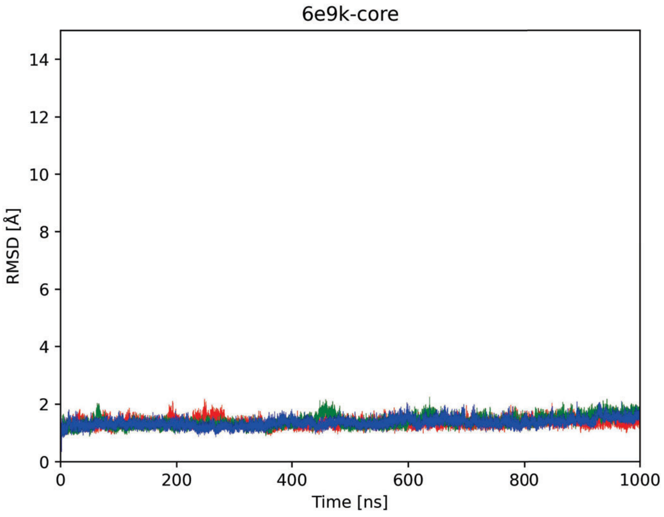
6e9k-core
RMSD [Å]
14
12
10
8
6
4
2
0
200
400
600
800
1000
Time [ns]

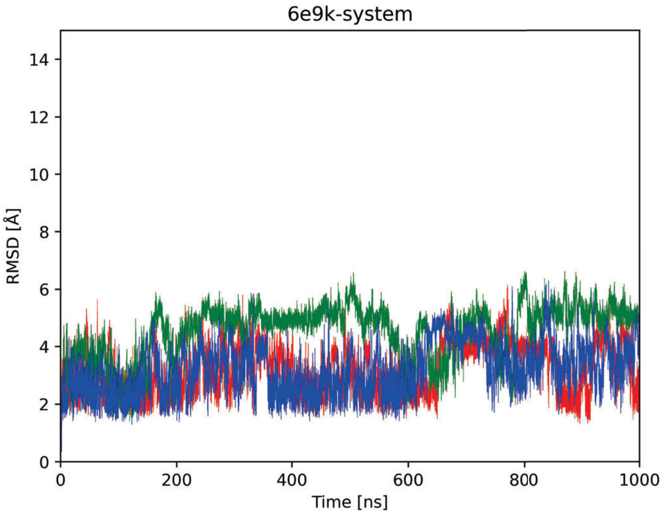
6e9k-system
RMSD [Å]
14
12
10
8
6
4
2
0
200
400
600
800
1000
Time [ns]

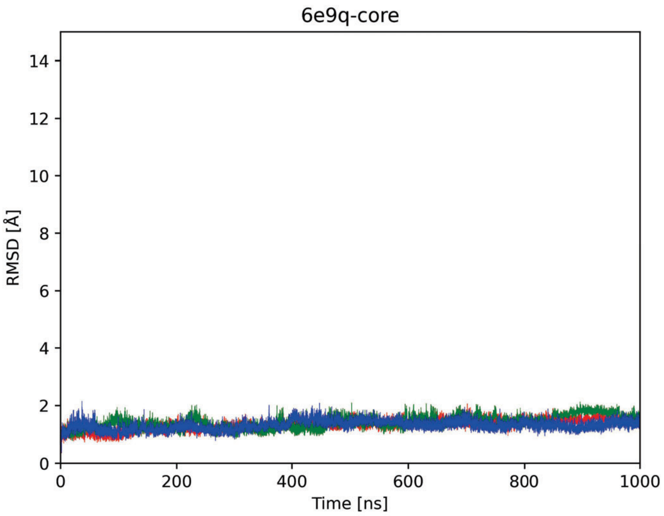
6e9q-core
RMSD [Å]
Time [ns]

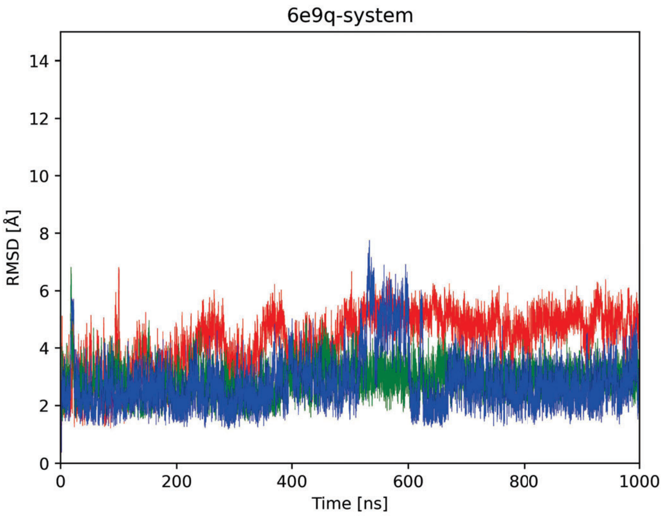
6e9q-system
RMSD [Å]
Time [ns]

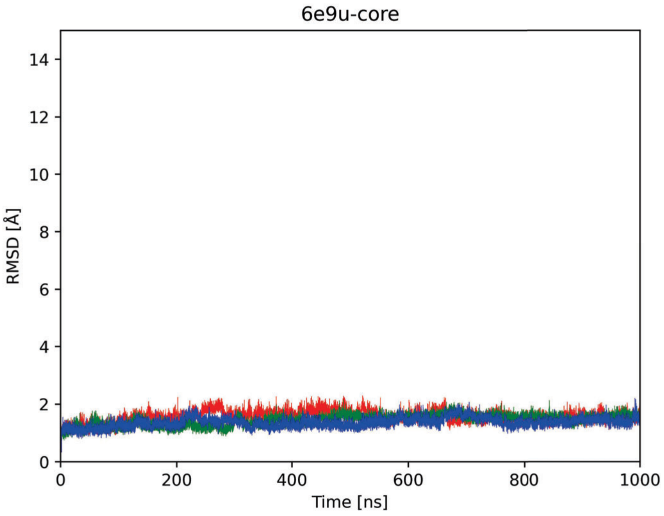
6e9u-core
RMSD [Å]
Time [ns]

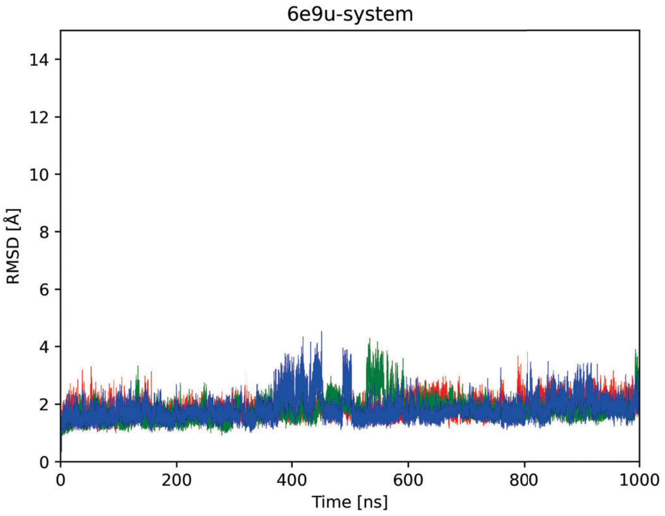
6e9u-system
RMSD [Å]
Time [ns]

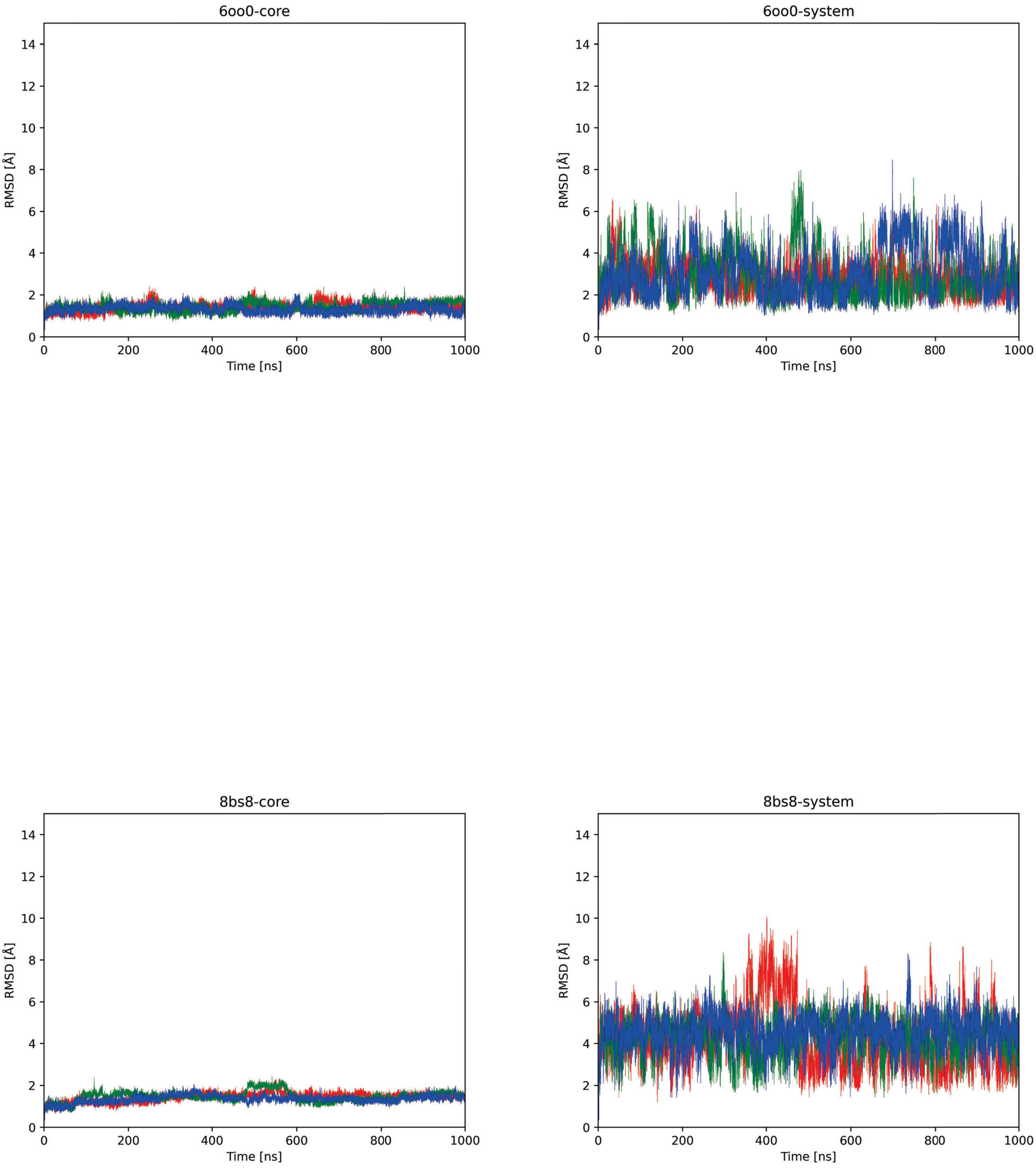

6oo0-core
6oo0-system
8bs8-core
8bs8-system
RMSD [Å]
Time [ns]
0
2
4
6
8
10
12
14
0
200
400
600
800
1000

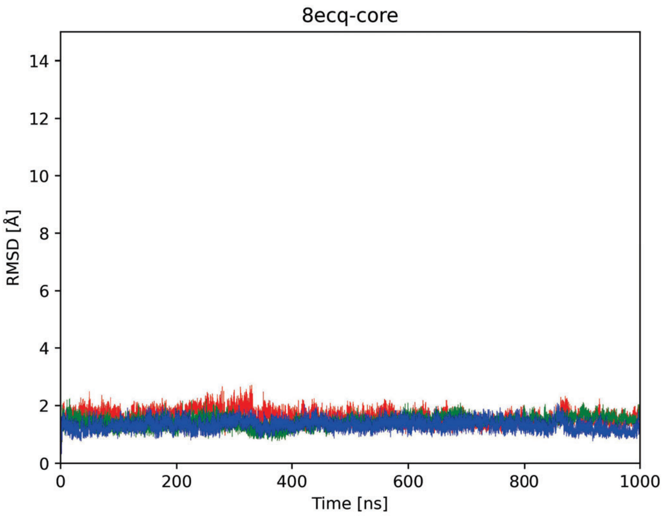
8ecq-core
RMSD [Å]
Time [ns]

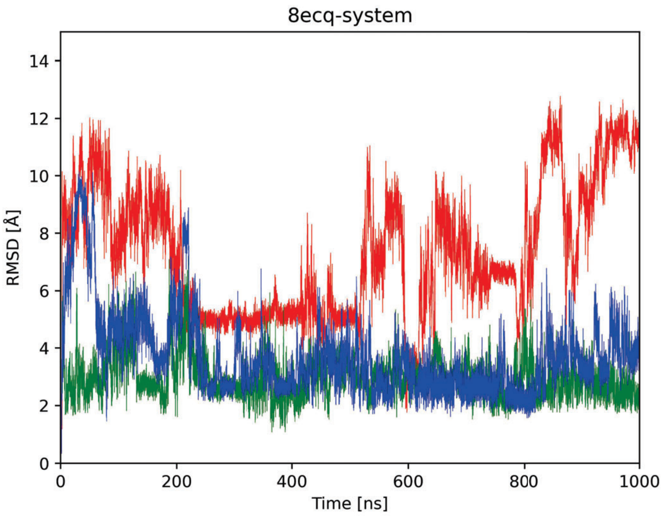
8ecq-system
RMSD [Å]
Time [ns]

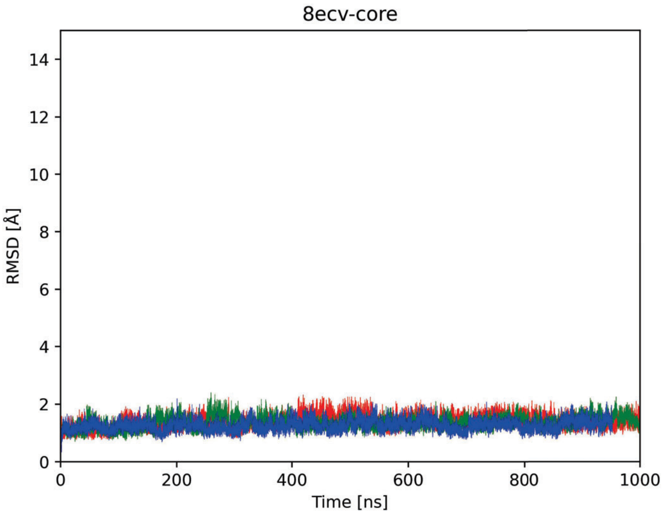
8ecv-core
RMSD [Å]
Time [ns]

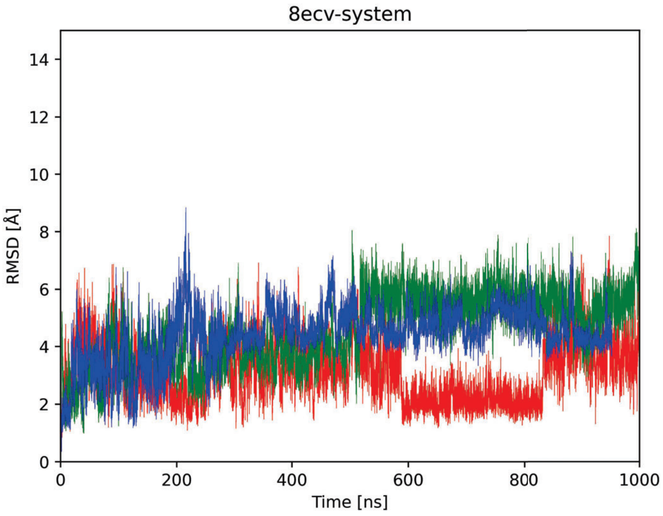
8ecv-system
RMSD [Å]
Time [ns]

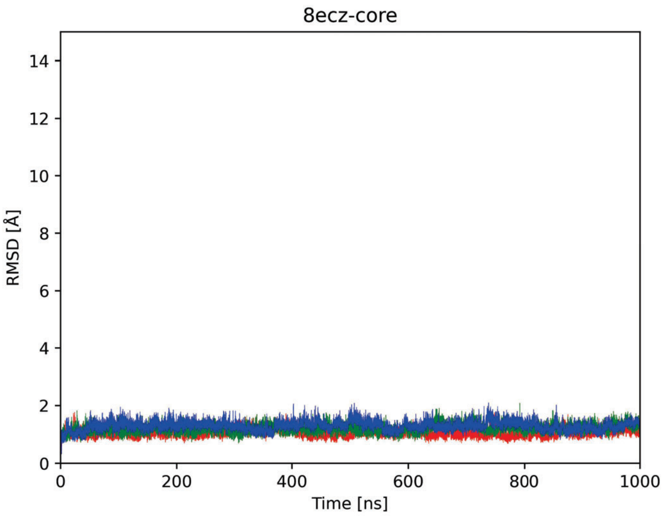
8ecz-core
RMSD [Å]
0
2
4
6
8
10
12
14
0
200
400
600
800
1000
Time [ns]

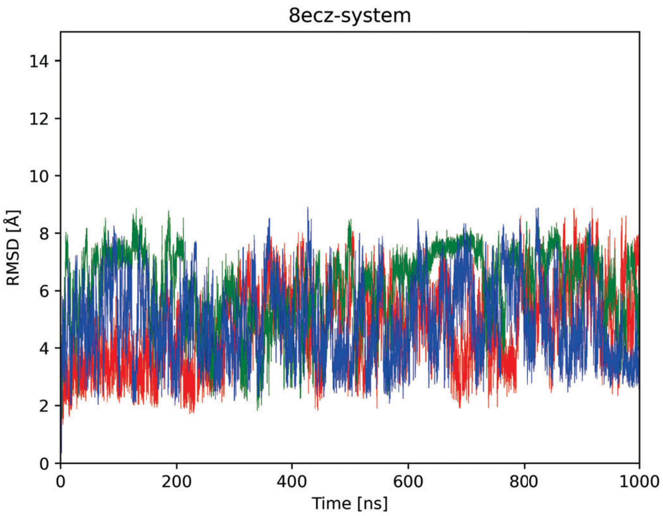
8ecz-system
RMSD [Å]
0
2
4
6
8
10
12
14
0
200
400
600
800
1000
Time [ns]

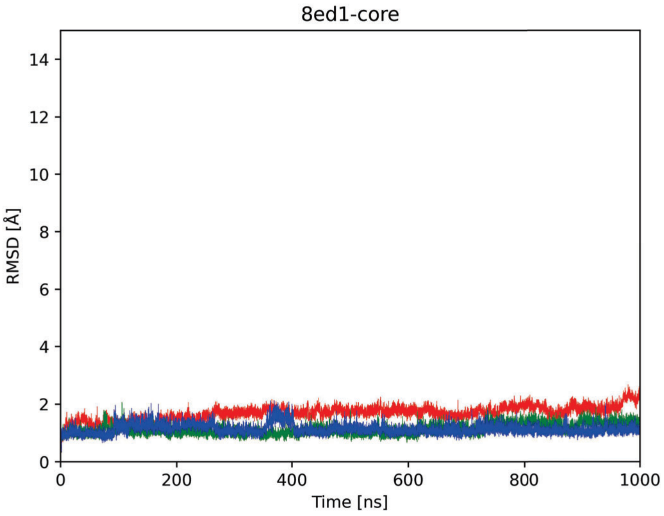
8ed1-core
RMSD [Å]
0
2
4
6
8
10
12
14
0
200
400
600
800
1000
Time [ns]

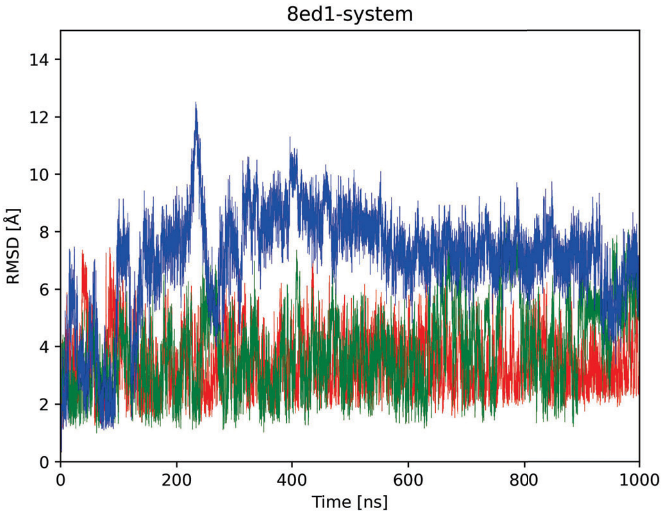
8ed1-system
RMSD [Å]
0
2
4
6
8
10
12
14
0
200
400
600
800
1000
Time [ns]

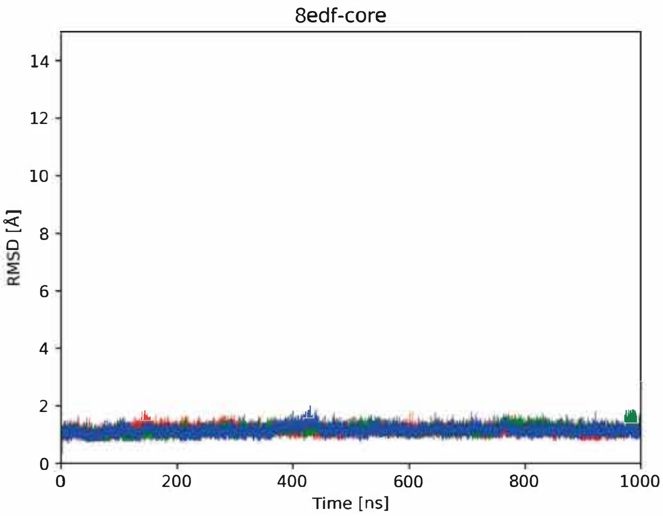
8edf-core
RMSD [Å]
Time [ns]

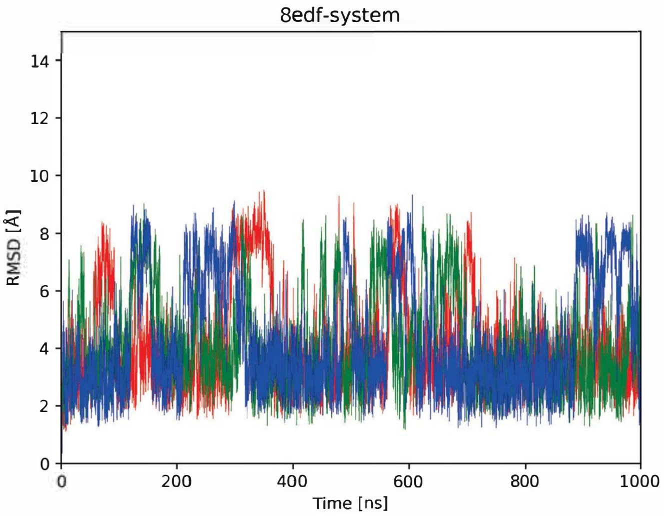
8edf-system
RMSD [Å]
Time [ns]
