# Supplementary material for: Comparative Molecular Dynamics Study of 19 Bovine Antibodies with Ultralong CDR H3
Source: Antibodies (Basel). 2025 Aug 13;14(3):70. doi: 10.3390/antib14030070 (PMC12371982; doi:10.3390/antib14030070)
Supplement: Supplementary file 1 [file antibodies-14-00070-s001.zip › Supplementary_Tables.pdf]

Supplementary Tables S1 – S3

Comparative molecular dynamics study of 19 bovine antibodies with ultralong CDR H3

Olena Denysenko [1], Anselm H. C. Horn [1,2], Heinrich Sticht [1,2*]

[1] Bioinformatics, Institute of Biochemistry, Friedrich-Alexander-Universität Erlangen-Nürnberg (FAU), Germany

[2] Erlangen National High Performance Computing Center (NHR@FAU), Friedrich-Alexander-Universität Erlangen-Nürnberg (FAU), Germany

* Correspondence: heinrich.sticht@fau.de

Table S1: RMSD values (in Å) for the individual simulation runs 19 investigated systems compared to the respective crystal structure. All values were averaged over the entire simulation time.

System	core domain (run A)	core domain (run B)	core domain (run C)	entire ulCAB system (run A)	entire ulCAB system (run B)	entire ulCAB system (run C)
4k3d	1.43	1.51	1.62	3.53	5.89	7.87
4k3e	1.14	1.00	1.30	3.48	2.83	3.04
5e99	1.11	1.36	1.24	4.21	7.53	5.99
5ijv	1.52	1.43	1.56	5.28	3.12	2.65
5ilt	1.08	1.08	1.18	7.59	7.38	3.79
6e8v	1.31	1.44	1.49	6.01	3.91	7.07
6e9g	2.03	1.64	2.09	6.40	6.48	5.95
6e9h	1.37	1.22	1.60	6.22	5.48	7.11
6e9i	1.68	1.27	1.32	3.64	3.59	3.04
6e9k	1.35	1.39	1.34	3.18	4.50	3.19
6e9q	1.33	1.41	1.30	4.29	2.85	2.77
6e9u	1.54	1.44	1.35	1.85	1.78	1.80
6oo0	1.40	1.42	1.31	2.77	2.93	3.06
8bs8	1.42	1.46	1.33	4.26	4.27	4.54
8ecq	1.58	1.43	1.31	7.30	2.96	3.72
8ecv	1.38	1.35	1.25	3.09	4.56	4.40
8ecz	1.06	1.19	1.29	4.75	6.11	4.85
8ed1	1.68	1.12	1.12	3.40	3.86	7.10
8edf	1.15	1.16	1.13	4.11	4.18	4.32

Table S2: Translational (transl.) and rotational (rot.) offset of the knob domain position for the individual simulation runs of the 19 investigated systems compared to the respective crystal structure. All values were averaged over the entire simulation time.

System	transl. [Å] (run A)	transl. [Å] (run B)	transl. [Å] (run C)	rot. [deg] (run A)	rot. [deg] (run B)	rot. [deg] (run C)
4k3d	13.62	22.10	28.35	43.14	64.63	99.58
4k3e	17.64	16.23	11.68	41.16	36.95	33.48
5e99	15.65	28.04	31.99	48.81	76.86	78.16
5ijv	17.00	13.86	12.20	57.39	39.37	34.42
5ilt	39.74	37.87	20.13	119.82	114.31	53.00
6e8v	24.96	12.32	29.78	72.27	44.94	120.62
6e9g	17.90	18.09	24.39	71.13	64.40	76.44
6e9h	27.25	24.59	29.06	99.67	80.76	99.94
6e9i	11.17	11.99	9.30	30.76	30.92	24.53
6e9k	10.28	11.05	11.23	29.00	45.77	36.27
6e9q	13.15	9.22	10.00	80.62	31.10	33.21
6e9u	2.81	3.46	3.02	14.60	14.21	11.79
6oo0	10.18	8.41	8.56	31.45	35.04	33.62
8bs8	13.84	14.65	14.59	41.83	41.59	45.94
8ecq	24.67	11.07	24.23	97.21	41.96	56.27
8ecv	10.09	16.36	21.38	30.23	65.46	90.31
8ecz	19.78	28.97	21.00	54.53	71.67	52.89
8ed1	12.08	16.04	18.96	38.20	52.27	71.68
8edf	12.08	11.63	12.38	47.03	47.56	50.17

Table S3: Van-der-Waals interaction energy (E_{vdW} in kcal/mol) between knob and core domain of the heavy chain for the 19 systems investigated. Values are average over the entire simulation time but shown separately for the three simulations runs. The last column gives the average value over all three simulation runs.

System	E_{vdW} (run A)	E_{vdW} (run B)	E_{vdW} (run C)	Average E_{vdW}
4k3d	0.00	0.00	0.00	0.00
4k3e	0.00	0.00	0.00	0.00
5e99	-0.01	-2.78	0.00	-0.93
5ijv	-0.15	-0.46	0.00	-0.21
5ilt	0.00	0.00	0.00	0.00
6e8v	-0.09	-0.01	-0.01	-0.03
6e9g	0.00	0.00	-3.78	-1.26
6e9h	0.00	0.00	0.00	0.00
6e9i	0.00	0.00	0.00	0.00
6e9k	0.00	0.00	0.00	0.00
6e9q	-0.03	-0.01	-0.09	-0.05
6e9u	-0.02	-0.02	-0.01	-0.02
6oo0	-0.01	0.00	0.00	0.00
8bs8	0.00	0.00	0.00	0.00
8ecq	-0.62	0.00	-0.02	-0.21
8ecv	0.00	-0.01	0.00	0.00
8ecz	0.00	0.00	0.00	0.00
8ed1	0.00	0.00	-0.20	-0.07
8edf	0.00	0.00	0.00	0.00
